# Mind the gap: a prospective observational study of interprofessional differences in ASA-PS assessments between surgeons and anaesthesiologists

**DOI:** 10.1186/s12871-026-03664-8

**Published:** 2026-02-04

**Authors:** Hanan El youzouri, Ananda Wagner, Guido Woeste, Ricardo Schnaudigel, Georgios Makridis, Kai Zacharowski, Philipp Helmer, Patrick Meybohm, Wolf O. Bechstein, Armin Wiegering, Eva Herrmann, Teresa Schreckenbach

**Affiliations:** 1 Department of General, Visceral, Transplantation, and Thoracic Surgery, Goethe-University Frankfurt/Main, Frankfurt University Hospital and Clinics, Theodor‑Stern‑Kai 7, 60590 Frankfurt/Main, Germany; 2 General practitioner’s office Dr. Stefan Rosenbrock, Altstadt 29, 64807 Dieburg, Germany; 3 Department of General and Visceral Surgery, AGAPLESION Elisabethenstift, Landgraf-Georg-Str. 100, Darmstadt, 64287 Germany; 4 Department of Anaesthesiology, Intensive Care Medicine and Pain Therapy, Goethe-University Frankfurt/Main, Frankfurt University Hospital and Clinics, Theodor-Stern-Kai 7, Frankfurt/Main, 60590 Germany; 5 Department of General and Visceral Surgery, St. Josefs-Hospital Wiesbaden, Beethovenstraße 20, Wiesbaden, 65189 Germany; 6 Department of Anaesthesiology, Intensive Care, Emergency and Pain Medicine, University Hospital Würzburg, Oberdürrbacher Str. 6, Würzburg, 97080 Germany; 7 Institute for Biostatistics and Mathematical Modeling, Goethe University Frankfurt am Mai, Theodor‑Stern‑Kai 7, Frankfurt/Main, 60590 Germany

**Keywords:** ASA-PS, Inter-rater reliability, Surgery, Anaesthesiology, Preoperative assessment

## Abstract

**Background:**

The American Society of Anaesthesiologists Physical Status (ASA-PS) classification system is the most widely used tool for estimating perioperative risk. Despite its widespread application, the ASA-PS is based solely on the subjective assessment of the patient’s clinical condition and comorbidities, which leads to considerable inter-rater variability. The aim of this study was to investigate interprofessional differences in ASA-PS scoring between surgeons and anaesthesiologists.

**Methods:**

This prospective observational study involved patients who were scheduled for elective or emergency surgery. The patients were independently evaluated using the ASA-PS classification by treating anaesthesiologists and surgeons with varying levels of experience. Following data collection, an interdisciplinary board comprising senior anaesthesiologists and surgeons retrospectively assigned reference ASA-PS scores. Inter-rater agreement was analysed using Cohen’s kappa (*κ*).

**Results:**

In total, 684 were included in the study. Surgeons assigned lower ASA-PS classes more frequently, with 16.81% of ratings corresponding to ASA-PS I and 52.05% to ASA-PS II, compared to 6.29% vs. 40.50% among anaesthesiologists. In contrast, anaesthesiologists more often classified patients as ASA-PS III (50.88%), than surgeons (28.51%). Agreement with the reference board was higher among anaesthesiologists (*κ* = 0.36) than among surgeons (*κ* = 0.21). The interprofessional agreement between surgeons and anaesthesiologists was low (*κ* = 0.25), which highlights the significant variability in clinical judgment between disciplines. ASA-PS assessments were more frequently performed by residents among surgeons than among anaesthesiologists (90.35% vs. 69.74%; *p* < 0.0001).

**Conclusion:**

The findings underscore the substantial inter-rater variability in ASA-PS scoring between surgical and anaesthesiological teams. While ASA-PS classification of ≥III remain clinically relevant and is widely used as a predictor for perioperative risk, the observed discrepancies may affect risk stratification in clinical practice. As most of ASA-PS assessments, especially in the surgeon group, were performed by residents, these results highlight the importance of structured training, and potentially supplementary objective tools to improve consistency and reliability in preoperative risk assessment.

**Trial registration:**

The study was registered at ClinicalTrials.gov (No. NCT02995499) and the German Clinical Trial register (No. 00011311, 12/2016).

**Supplementary Information:**

The online version contains supplementary material available at 10.1186/s12871-026-03664-8.

## Background

Annually, more than 310 million major surgeries are performed worldwide [1]. Assessment prior to surgery is mandatory for each individual patient. The most common tool for assessment of surgery-related risk is the American Society of Anaesthesiologists Physical Status Classification System (ASA-PS classification), which relies on a non-objective assessment of the patient by the treating anaesthesiologist [2]. This assessment contributes to perioperative risk stratification and may influence intraoperative management procedures such as invasive blood-pressure monitoring as well as decisions regarding postoperative ICU observation, in conjunction with surgical risk and other clinical factors. Thus, ASA-PS assessment has a major impact for all operative procedures as it helps to predict increased risk of perioperative complications [3–5]. 

The ASA-PS classification is a six-point score that is used to assess the physical status of patients and their risk of perioperative mortality [6, 7]. It is assigned through a physician’s clinical evaluation of a patient’s status based on physical examination and medical history without further assessments like the ‘Timed Up and Go Test’ or other frailty scoring systems [8]. The ASA-PS classification is easily administered in daily practice but is often criticised for its tendency toward interindividual differences due to high subjectivity and varying levels of clinical experience of the assessors. These factors result in interindividual variability, which has been examined in multiple studies worldwide [9–11]. Studies have examined the inter-rater reliability of the ASA-PS classification between different raters as a statistical measure to address this inconsistency [9, 11, 12]. Overall, high variability of the inter-rater reliability has been demonstrated in the literature with kappa values (*κ*) between − 0.042 and 0.863, which correspond to a range spanning from no agreement to almost complete agreement [12–14]. 

The ASA-PS score is usually determined by anaesthetists. Assessments of operability by surgeons are usually based on the patient’s clinical impression or frailty assessment and is only rarely quantified as an ASA-PS score [13]. In a preoperative setting, patient contact patterns differ substantially between surgeons and anaesthesiologists. Surgeons typically engage with patients on multiple occasions during surgical workup, which may provide a more longitudinal view of the patient’s condition [14]. 

In contrast, anaesthesiologists often assess patients in a single preoperative consultation that focuses primarily on the perioperative period-related consideration [15]. These differing levels of interaction may influence the perception of perioperative risk and could contribute to variability in ASA-PS classification between the two specialties [16]. A study using simulated patient cases showed that non-anaesthesiologist clinicians, including surgeons, tend to assign lower ASA-PS scores compared to anaesthesiologists, which could potentially lead to an underestimation of surgical risk [16]. 

Based on this literature, we hypothesized that ASA-PS assessments performed by surgeons differ systematically from those assigned by anaesthesiologists. The primary aim of this study was to assess the inter-rater reliability of the ASA-PS classification between anaesthesiologists and general surgeons, and its reproducibility across disciplines using real-world data in a prospective setting. The secondary aim was to evaluate the degree of discrepancy between ASA-PS classifications assigned by anaesthesiologists and general surgeons compared with the ASA-PS classifications assigned by a consensus from an interdisciplinary panel, in relation to the individual assessors’ professional experience.

## Methods

### Design

**Fig. 1:**
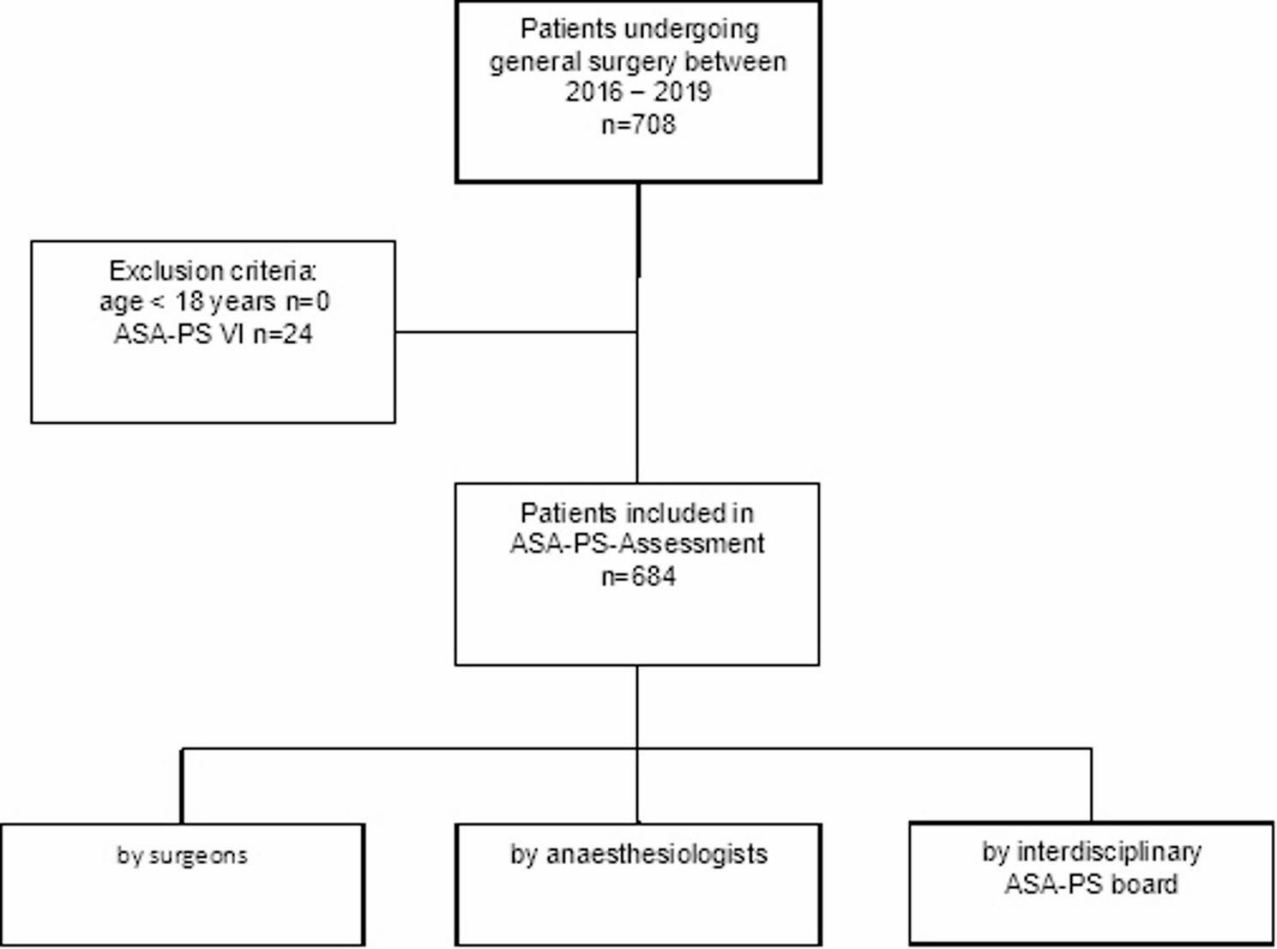
Flow Chart

This prospective observational study included patients receiving general anaesthesia while undergoing general surgery in an elective or emergency setting at the Department of General, Visceral, and Transplant Surgery at a single tertiary care centre in Germany between August 2016 and August 2019. All patients aged ≥ 18 years undergoing surgery in this period with an ASA-PS between I and V were included. Exclusion criteria were age under 18 years or ASA-PS VI (Fig. 1). Patient selection followed a convenience sampling approach based on the availability of physicians to perform the ASA-PS classification. Each patient was independently assessed by an anaesthesiologist during the pre-anaesthesia risk assessment visit and by the operating surgeon. Depending on the clinical setting, both residents and board-certified specialists participated in the ASA-PS assessments. Both the anaesthesiologists and surgeons were blinded to the other’s evaluation. Unlike the surgeons, the anaesthesiologists did not know which patients were included in the study. Additionally, an interdisciplinary board consisting of surgeons and anaesthesiologists retrospectively assigned an ASA-PS score based on the available electronic health record (EHR). ASA physical status was assessed in accordance with ASA guidelines and reflected the patient’s comorbidities and preoperative overall systemic condition. The underlying surgical diagnosis was not taken into account unless it resulted in systemic deterioration.

The study was conducted in accordance with the Declaration of Helsinki and was approved by the Ethics Committee of the Department of Medicine at the Johann Wolfgang Goethe University Frankfurt (No. 405/16, 10/2016). Written informed consent was obtained from all patients before inclusion in the study. The study was registered at ClinicalTrials.gov (No. NCT02995499) and the German Clinical Trial register (No. 00011311, 12/2016). This study was conducted and reported in accordance with the Strengthening the Reporting of Observational Studies in Epidemiology (STROBE) guidelines.

### Data collection

Demographic data were obtained from the EHR and included sex, age, body mass index (BMI), clinical risk factors (smoking, alcohol), comorbidities, and previous surgeries. The data recorded pertaining to the index surgery included the type, duration, and urgency (emergency vs. elective surgery), as well as postoperative complications according to the Clavien–Dindo Classification (CDC), which were collected to descriptively characterise the postoperative course of the study population and not as an outcome related to the primary study objectives [17]. The main diagnoses and the type of surgery performed were coded according to International Statistical Classification of Diseases and Related Health Problems (ICD10) classification and International Classification of Procedures in Medicine (ICPM). All personal data were pseudonymized and entered into a Microsoft Excel^®^ database.

### ASA-PS assessment

The ASA-PS classification was determined preoperatively by both the anaesthesiologists during the pre-anaesthesia risk-assessment visit, and by the surgeon, according to the official ASA Physical Status Classification System definitions. In contrast to the surgeons, the anaesthesiologists were blinded for patients’ participation in the study. Blinding of surgeons to study participation was not feasible, as ASA-PS classification is not routinely assigned by surgeons in standard clinical practice and was performed specifically for the purpose of this study. In contrast, anaesthesiologists assigned the ASA-PS score as part of routine preoperative care, and these assessments were subsequently used for study analysis. In addition, without prior inspection of the preoperatively documented ASA-PS class or perioperative course, a further ASA-PS assessment was carried by an interdisciplinary board that included a senior surgeon and a senior anaesthesiologist. The interdisciplinary board assessment was pre-planned as part of the study design. Its purpose was to provide a consensus-based reference ASA-PS classification by integrating the perspectives of both a senior surgeon and a senior anaesthesiologist based on comprehensive EHR review. The board was not intended to represent a definitive gold standard, but rather a structured reference to facilitate comparison between disciplines.

### Endpoints and sample-size calculation

The primary endpoint was defined as the inter-rater reliability between the ASA-PS score assigned by the three rater groups (surgeons, anaesthesiologists, and the interdisciplinary board). The primary outcome variable was the ASA-PS classification assigned by each rater group. The secondary endpoints were the inter-rater reliability within each profession and the board and between the ASA-PS status and the level of professional experience. ASA-PS classifications were analysed on an individual category level to allow a detailed assessment of inter-rater variability. The sample size calculation was based on precision considerations for the primary agreement measure, Cohen’s weighted kappa. An expected kappa value of 0.53 was assumed based on a previously published multicentre study investigating inter-rater agreement of ASA physical status classification [11]. Assuming a conservative standard deviation of κ of 1.0 and targeting a two-sided 95% confidence interval with a maximum width of 0.15, a total sample size of 683 patients was required using established methods for confidence interval estimation of kappa statistics.

### Statistics

The raw data were collected and formatted in Microsoft Excel^®^ to enable statistical evaluation using BiAS^®^. BiAS is a Windows-based statistical program that offers a comprehensive suite of analytical tools, including descriptive statistics, parametric and non-parametric hypothesis tests, regression methods, power and sample size calculations, and graphical output suitable for scientific reporting [18]. 

Unless otherwise noted, the exploratory statistics are shown as integer values with mean and percentage values, as well as median and standard deviations. Decimals were rounded to the second decimal place. Categorical variables were compared between groups using the chi-square test. The null hypothesis for these analyses was defined as no difference in the distribution of categorical variables between the compared groups. A value of *p ≤* 0.05 was considered statistically significant. 95% CIs were used for all test procedures.

Inferential statistical analyses focused on inter-rater reliability. Agreement between two raters was assessed using Cohen’s kappa (κ), while agreement among more than two raters was evaluated using Fleiss’ kappa. For these analyses, the null hypothesis was defined as no agreement beyond chance (κ = 0). Statistical significance of κ values was determined using corresponding hypothesis tests and 95% confidence intervals. Potential sources of bias include selection bias due to the convenience sampling approach and observer bias related to the subjective nature of the ASA-PS assessment. Blinding between raters and the use of an interdisciplinary reference board were applied to mitigate these effects.

Inter-rater reliability describes the degree of agreement in assessment results between different raters and can be estimated using Cohen’s kappa (*κ*) for two raters and Fleiss’ kappa for more than two raters [19, 20]. The assessment of inter-rater reliability using kappa was based on Landis and Koch’s assessment with *κ* < 0 indicating “poor” reliability, *κ* < 0.20 indicating “slight”, *κ* = 0.21*–*0.40 indicating “fair”, *κ* = 0.41*–*0.60 indicating “moderate”, *κ* = 0.61*–*0.80 indicating “substantial”, and *κ* = 0.81*–*1.00” indicating “(almost) perfect agreement” [21]. A Sankey flow diagram was obtained using the software RawGraphs 2.0 [22]. 

## Results

### Demographics

Among the included 684 patients, the mean age was 58.1 ± 16.26 years. There were 389 male (56.87%) and 295 female (43.13%) patients. 51 (7.46%) patients required emergency surgery. Fifteen of the 684 patients (2.19%) died within 30 days following surgery. Further details are shown in Table 1. An analysis describing the relevant comorbidities corresponding to the ASA-PS board classification is provided in Supplementary Table 1.

**Table 1:** Demographics and baseline data according to final ASA-PS board classification; percentages given in relation to the respective category

	ASA-PS I n = 37	ASA-PS II n = 356	ASA-PS III n = 264	ASA-PS IV n = 27	Total n = 684
Age ≥ 65 years	2 (5%)	131 (37.08%)	125 (47.35%)	15 (55.56%)	273 (39.91%)
Male	21 (56.76%)	172 (48.31%)	177 (67.05%)	19 (70.37%)	389 (56.87%)
Smoker	2 (5%)	85 (23.88%)	71 (26.89%)	7 (25.93%)	165 (24.3%)
Previous smoker	0	20 (5.62%)	23 (8.71%)	5 (18.52%)	48 (7.02%)
Alcohol abuse	2 (5%)	14 (3.93%)	53 (20.08%)	1 (3.7%)	70 (10.3%)
Previous alcohol abuse	0	2 (0.56%)	23 (8.71)	3 (11.11%)	28 (4.09%)
Intravenous drug abuse (current and past)	0	0	6 (2.27%)	0	6 (0.88%)
BMI					
BMI 25–29 kg/m²	12 (32.4%)	141 (39.61%)	88 (33.33%)	10 (37.03%)	251 (36.7%)
BMI 30–34 kg/m²	1 (2.7%)	48 (13.62%)	40 (15.15%)	3 (11.11%)	92 (13.45%)
BMI 35–39 kg/m²	0	20 (5.63%)	8 (3.03%)	1 (3.7%)	29 (4.24%)
BMI ≥ 40 kg/m²	0	1 (0.28%)	14 (5.3%)	1 (3.7%)	16 (2.34%)
BMI ≤ 17 kg/m²	0	4 (1.12%)	7 (2.65%)	0	11 (1.61%)
CDC					
CDC 0	31 (83.78%)	214 (60.11%)	137 (52.29%)	14 (56%)	396 (57.89%)
CDC I–II	4 (10.81%)	64 (17.98%)	64 (24.43%)	6 (24%)	138 (20.18%)
CDC III–V	2 (5.41%)	74 (20.79%)	61 (23.28%)	5 (20%)	142 (18.13%)
CDC V	1 (2.7%)	8 (2.25%)	6 (2.29%)	0	15 (2.19%)
Previous surgery	18 (48.65%)	186 (52.25%)	119 (45.08%)	19 (70.37%)	342 (50%)
Emergency surgery	9 (24.32%)	21 (5.9%)	17 (6.44%)	4 (14.81%)	51 (7.46%)

ASA-PS (American Society of Anaesthesiologists Physical Status Classification); BMI (body mass index)

### Participating raters

Overall, 618 (90.35%) of the surgical assessments were made by residents, and 66 (9.65%) were made by board-certified surgeons. Among anaesthesiologists, 477 (69.74%) evaluations were done by residents, and 207 (30.26%) were done by board-certified anaesthesiologists. This difference between the groups was statistically significant (*p <* 0.0001).

The ASA-PS board assessment was carried out by two consultant surgeons of general and visceral surgery (HE, GW; 13 and 18 years of professional experience, respectively) and two consultant anaesthesiologists (PM, PH; 16 and 11 years of professional experience, respectively).

### Surgical details and postoperative complications

The operations performed were subdivided into different categories based on the organ system (Table 2). The category “other” includes sarcoma resections, lymphadenectomies, or the application of negative-pressure wound-therapy systems. A total of 369 (53.95%) procedures were carried out on patients with cancer.

**Table 2:** Frequency of conducted surgery types

Organ system	Absolute number	Relative frequency
Thyroid/parathyroid gland	30	4.39%
Liver	130	19.01%
Bile duct	10	1.46%
Gallbladder	45	6.58%
Pancreas	77	11.26%
Upper gastrointestinal tract	44	6.43%
Lower gastrointestinal tract/colon/rectum	117	17.11%
Gastrointestinal tract/small intestine	20	2.92%
Appendix	20	2.92%
Anus	24	3.51%
Adrenal gland	21	3.07%
Spleen	4	0.58%
Abdominal Wall (hernia)	53	7.75%
Multivisceral resection	1	0.15%
Organ Transplantation	5	0.73%
Others	43	6.29%
Diagnostic laparoscopy	16	2.34%
Laparotomy (without specific organ-system assignment)	24	3.51%

Postoperative complications were recorded to descriptively characterise the postoperative course of the study population. The CDC result was available for 676 (98.83%) patients. For 8 (1.17%) patients, no assessment of the postoperative course could be made due to incomplete data. Supplementary Table 2 gives the details of postoperative complications.

### ASA-PS assessment and inter-rater reliability

Surgeons more frequently assigned lower ASA-PS categories, classifying 68.86% of patients as ASA-PS I–II and 31.15% as ASA-PS III–V. In contrast, anaesthesiologists classified 46.79% of patients as ASA-PS I–II and 53.22% as ASA-PS III–IV. Anaesthesiologists showed a higher overall agreement rate of 63.02% with the ASA board’s classifications than the surgeons (52.34%). The frequencies of the ASA-PS assessments by the different disciplines and ASA-PS board are shown in Table 3.

**Table 3:** Distribution of ASA-PS assessments made in absolute and relative numbers and distribution of the degree of agreement between the surgeons, anaesthesiologists and the interdisciplinary board

ASA-PS	Surgeons		Anaesthesiologists		ASA-PS Board	
I	115 (16.81%)		43 (6.29%)		37 (5.41%)	
II	356 (52.05%)		277 (40.5%)		356 (52.04%)	
III	195 (28.51%)		348 (50.88%)		264 (38.6%)	
IV	17 (2.49%)		16 (2.34%)		27 (3.95%)	
V	1 (0.15%)		0		0	
Match Rate with Anaesthesiologist						
	ASA-PS I	ASA-PS II		ASA-PS III		ASA-PS IV
Surgeons	30 (26.09%)	179 (50.28%)		156 (80.00%)		2 (11.76%)
Match Rate with ASA-PS board decisions						
	ASA-PS I	ASA-PS II		ASA-PS III		ASA-PS IV
Surgeons	29 (78.38%)	215 (60.39%)		109 (41.29%)		5 (18.52%)
Anaesthesiologists	22 (59.46%)	204 (57.3%)		198 (75.0%)		7 (25.93%)

ASA-PS (American Society of Anaesthesiologists Physical Status Classification System)

**Fig. 2:**
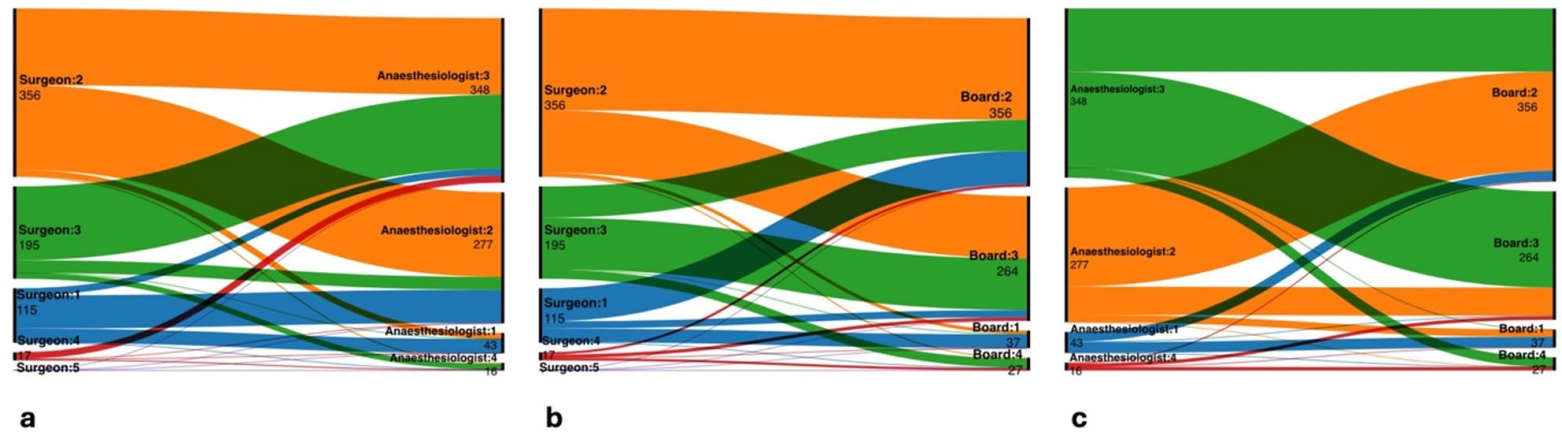
Sankey flow diagram of ASA-PS between surgical, anaesthesiological, and board evaluations. The diagram illustrates interprofessional variation in ASA scoring across the three rater groups. Each node represents an assigned ASA class (I–V), while the links between nodes indicate the frequency of matching or diverging classifications. The left-to-right sequence reflects assessments made by surgeons and anaesthesiologists (**a**), surgeons and interdisciplinary board (**b**), and anaesthesiologists and interdisciplinary board (**c**). Line thickness is proportional to the number of patients assigned each combination

The evaluation of the inter-rater reliability using Fleiss’ kappa showed sufficient agreement between the three rater groups with a kappa value of *κ* = 0.28 (CI 0.21–0.34; *p <* 0.0001). The inter-rater reliability between the surgeons and anaesthesiologists showed fair agreement between both groups with a kappa value of *κ* = 0.25 (*p <* 0.0001, CI 0.19–0.30). Between surgeons and the ASA-PS board, the inter-rater reliability showed fair agreement with *κ* = 0.21 (*p <* 0.0001, CI 0.15–0.27). Between the anaesthesiologists and the board, inter-rater variability showed a kappa value of *κ* = 0.36 (*p <* 0.0001, CI 0.30–0.43). Table 4 summarize these findings and Fig. 2 shows the Sankey flow diagram of the ASA-PS evaluations between surgeons, anaesthesiologists, and the board.

**Table 4:** Inter-rater reliability of ASA-PS classification between rater groups

	κ (Cohen’s/Fleiss’)	95% CI	*p*-value	Interpretation^*^
Surgeons vs. Anaesthesiologists	0.25	0.19–0.30	< 0.0001	Fair agreement
Surgeons vs. ASA-PS board	0.21	0.15–0.27	< 0.0001	Fair agreement
Anaesthesiologists vs. ASA-PS board	0.36	0.30–0.43	< 0.0001	Fair agreement
Overall	0.28^†^	0.21–0.34	< 0.0001	Fair agreement

^*^Interpretation according to Landis and Koch [21]

^†^Fleiss’ κ

## Discussion

This study assessed differences in the ASA-PS classification between treating disciplines. More than 80% of the patients assessed by all three groups fell into ASA-PS categories II and III. The surgeons assessed 80.56% of the patients as ASA-PS II or III, whereas 90.64% and 91.83% of patients were classified as ASA-PS II–III by the ASA-PS board and anaesthesiologists, respectively. This result is in line with previous studies, which have also criticised the insufficient discriminatory power between these two classes [8, 11]. 

Compared to anaesthesiologists, surgeons tended to rate patients as healthier according to ASA-PS classification. The inter-rater reliability between surgeons, anaesthesiologists, and the ASA-PS board yielded Cohen’s κ values of 0.21 (surgeons vs. board), 0.36 (anaesthesiologists vs. board), and overall κ = 0.25 between surgeons and anaesthesiologists. According to Landis and Koch, these values fall into the “fair” agreement range (0.21–0.40), with descriptively higher agreement observed for anaesthesiologists compared to surgeons [21]. Although anaesthesiologists showed numerically higher κ values, all inter-rater reliability estimates fell within the same “fair” agreement category according to Landis and Koch, and should therefore not be interpreted as indicating substantial inter-group differences.

A similar study by Curatolo et al. reported higher agreement between anaesthesiologists and a reference standard than between surgeons and the reference, consistent with the pattern observed in our study [16]. This further supports the point that, although profession affects scoring tendencies, agreement quality remains at best moderate.

It is important to note that the majority of ASA-PS assessments in both groups were performed by residents, and the present findings therefore primarily reflect ASA-PS assignment in a training-level clinical setting and should be interpreted accordingly. ASA-PS classification is an integral part of anaesthesiology training and routine clinical practice, which may contribute to closer alignment with the interdisciplinary ASA-PS board. In contrast, ASA-PS classification is less relevant in the surgeons’ daily clinical routine, so surgeons were provided with the ASA-PS example catalogue to support their assessments [23]. Differences in routine exposure and formal training in ASA-PS classification, rather than overall clinical experience alone, may therefore contribute to the observed interprofessional variability. The use of a retrospective interdisciplinary board as a reference for ASA-PS classification warrants also consideration. The board assessment was pre-planned and intended to provide a structured, consensus-based reference by integrating surgical and anaesthesiological perspectives based on EHR review. However, as the board had no direct patient contact and performed its assessment retrospectively, it should not be regarded as a definitive gold standard but rather as a pragmatic reference framework for interprofessional comparison.

The example catalogue is comprehensive, but the ASA-PS postulates that while it is intended to provide assistance, it does not cover all criteria [6, 23]. This aspect is reflected in the fact that the surgeons’ assessment shows less agreement with the reference standard despite having seen the ASA-PS catalogue. This result highlights the major significance of clinical experience in ASA-PS assessments. Furthermore, ASA-PS classification is an integral part of clinical training for anaesthesiologists starting on day one of their residence [9, 24]. 

Other studies consistently report that inter-rater reliability for ASA-PS varies widely, ranging from fair to very good, but tends to fall predominantly in the moderate category (κ = 0.4–0.6) [9, 12, 25]. Parenti et al. found most studies to demonstrate moderate agreement, with a few showing fair or even good reliability [24]. Sankar et al. similarly report moderate inter-rater reliability in clinical practice (κ = 0.53–0.61) [10]. One possible explanation for the lower κ values observed in the present study is the high proportion of ASA-PS assessments performed by residents. By reflecting routine clinical practice with assessors at different stages of training, our study captures a broader range of clinical judgment, which may result in lower inter-rater agreement.

A major advantage of ASA-PS classification is its simplicity, which contributes to its widespread use. History and clinical examination alone are sufficient for elicitation, and no further instrumental or laboratory diagnostic tests are required. The score has also been used in policy making, performance evaluation, resource allocation, and reimbursement of anaesthesia services and is frequently cited in clinical research.

However, this ease also presents major disadvantages and limitations. As early as 2002, Mak et al. stated that “a more specific and precise” system is urgently needed [25]. 

Reasons for inter-rater discrepancies include varying levels of professional experience, an insufficient example catalogue, and ambiguity in how multimorbidity should be classified [9, 26, 27]. Additionally, studies have shown that less experienced clinicians may rely more heavily on the provided examples, while senior clinicians might default to personal clinical judgment, potentially reducing standardization [9, 26, 28]. In summary, the study confirms that ASA-PS score assignment is profession-dependent, but also reveals that, regardless of profession, inter-rater agreement with a gold standard remains only fair to moderate.

### Limitations

In contrast to the anaesthesiological and surgical assessment, the interdisciplinary board did not have direct patient access but only had the EHR available for assignment of the ASA-PS score. The board assessment was performed retrospectively and did not constitute a prospective reference standard, which may limit comparability with the prospective clinical assessments. In addition, the ASA-PS board consisted of only four physicians, and their assessment of the ASA-PS score is also subjective. Surgeons may be more optimistic or less risk averse compared to anaesthesiologists. The underestimation of ASA-PS status by surgeons in this study is even more notable since they had been provided with an ASA-PS form explaining the grading, in contrast to anaesthesiologists, who are assumed to use the ASA-PS classification as a daily tool. Differences in blinding procedures between disciplines represent an additional limitation. While anaesthesiologists were unaware of study participation because ASA-PS classification is part of routine preoperative assessment, surgeons could not be blinded, as they do not routinely assign ASA-PS scores outside a study setting. The ASA-PS classification was initially introduced to assess the risk of anaesthesia, not the risk of surgery. Given the high proportion of patients with major surgery, the risk of complex surgery may override the risk of anaesthesia [29]. 

Furthermore, a large proportion of ASA-PS assessments were performed by residents. No sensitivity analyses stratified by level of clinical experience were conducted. However, this reflects routine clinical practice in many institutions and was intentionally accepted to enhance the external validity of the study. The potential influence of assessor experience on ASA-PS assignment should therefore be considered when interpreting the results.

Finally, in some healthcare systems, ASA classification may have implications for reimbursement, which could influence scoring behaviour. However, in the German DRG-based system, ASA physical status does not directly affect reimbursement.

### Strengths

To our knowledge, this is the first time that the ASA-PS classification has been prospectively studied regarding inter-rater variability across different specialties (i.e. surgeons vs. anaesthesiologists) with real patients in everyday clinical practice. A case-number calculation was performed, and more than 600 patients were included in the study. The surgeons and the interdisciplinary board were blinded according to the anaesthesiological evaluation to ensure unbiased classification of patients.

## Conclusions

The ASA-PS system remains a widely accepted and practical tool for preoperative risk stratification and is valued particularly for its simplicity and accessibility. Our findings demonstrate relevant interprofessional variability in ASA-PS assessments, reflecting the inherent subjectivity of the classification and differing clinical perspectives between surgeons and anaesthesiologists. This variability highlights the need for increased awareness of potential biases in ASA-PS scoring and support the use of structured training or complementary approaches to improve consistency and reproducibility.

While ASA-PS remains clinically useful, future strategies may explore supplementary tools to support risk assessment and standardize its application across disciplines.

## Electronic Supplementary Material

Supplementary Material 1.

Supplementary Material 2.

## Data Availability

The datasets generated and analysed in the current study are not publicly available due to the Ethics Committee restrictions but are available from the corresponding author upon reasonable request.

## Declarations

KZ has received honoraria for participation in advisory board meetings for Haemonetics, Boston (USA) and Vifor, Munich (Germany) and received speaker fees from CSL Behring, Hattersheim (Germany), Masimo, Irvine (USA), Pharmacosmos, Copenhagen (Denmark), Boston Scientific, Düsseldorf (Germany), Salus, Bruckmühl (Germany), iSEP, Nantes (France), Edwards, Munich (Germany), Hemosonics, Asnières-sur-Seine (France) and GE Healthcare, Düsseldorf (Germany). He is the Principal Investigator of the EU-Horizon 2020 project ENVISION (Intelligent plug-and-play digital tool for real-time surveillance of COVID-19 patients and smart decision-making in Intensive Care Units) and Horizon Europe 2021 project COVend (Biomarker and AI-supported FX06 therapy to prevent progression from mild and moderate to severe stages of COVID-19). Partner for EU Horizon 2023 project EDiHTA.

KZ leads as CEO the Christoph Lohfert Foundation, Hamburg (Germany) as as well as the Health, Patient Safety & PBM Foundation, Bad Homburg (Germany).

PH received a research award from Vogel-Foundation and is a member of the Clinician Scientist Programme, Wuerzburg.

WOB received consultation fees from Terumo. He also received honoraria for lectures, presentations, speakers bureaus, manuscript writing or educational events from AOK, Astellas, BDC, Chiesi, Deutscher Ärzteverlag, Else-Kröner Stiftung, Gesundheit ÖsterreichGmbH, Gore Deutschland, Hessisches Ministerium für Soziales und Intergration, Landesärztekammer Hessen, Landesärztekammer Saarlan, MCIAcademy, Sanofi-Aventis. Furthermore, he received support for attending meetings from Astellas, Charité Berlin, Chiesi, DGAV, DGCh, Deutsche Gesellschaft für Senologie, DeutscherÄrzteverlag, Deutscher Krebskongress, Gore Deutschland, HopScotch Paris, Interplan, Landesärztekammer Hessen, MCN, medupdate GmbH, Sanofi-Aventi; participate on a Data Safety Monitoring Board or Advisory Board for Terumo and is a past President of Deutsche Gesellschaft fürAllgemein-und Viszeralchirurgie (DGAV).

TS received Travel Grants from the Japanese Surgical Society and from German Society for General and Visceral Surgery.

## Abbreviations

ASA: PS American Society of Anaesthesiologists Physical Status
ICU: Intensive Care Unit
EHR: Electronic Health Record
CDC: Clavien–Dindo Classification
DRG: Diagnosis Related Groups
ICD10: International Statistical Classification of Diseases and Related Health Problems
ICPM: International Classification of Procedures in Medicine
BMI: Body Mass Index
EF: Ejection Fraction
ICD: Implantable Cardioverter-Defibrillator
COPD: Chronic Obstructive Pulmonary Disease
OSAS: Obstructive Sleep Apnoea Syndrome
CKD: Chronic Kidney Disease
IBD: Inflammatory Bowel Disease
HIV: Human Immunodeficiency Virus
