# Supplementary material for: Mind the gap: a prospective observational study of interprofessional differences in ASA-PS assessments between surgeons and anaesthesiologists
Source: BMC Anesthesiol. 2026 Feb 4;26:143. doi: 10.1186/s12871-026-03664-8 (PMC12934034; doi:10.1186/s12871-026-03664-8)
Supplement: Supplementary file 1 — Supplementary Material 1. [file 12871_2026_3664_MOESM1_ESM.docx]

**Supplementary Table 1** Relevant concomitant disease according to final ASA-PS board classification; percentages given in relation to the respective category.

| **Co-morbidity** | **ASA-PS I** | **ASA-PS II** | **ASA-PS III** | **ASA-PS IV** | **Total** |
| --- | --- | --- | --- | --- | --- |
| **Number of patients** | 37 | 356 | 264 | 27 | 684 |
| **Metabolic syndrome** | 0 | 9 (2.53%) | 19 (7.2%) | 3 (11.11%) | 31 (4.53%) |
| **Coronary artery disease** | 0 | 8 (2.25%) | 77 (29.17%) | 13 (48.15%) | 98 (14.33%) |
| **Myocardial infarction < 3 months** | 0 | 0 | 3 (1.14%) | 1 (3.7%) | 4 (0.58%) |
| **Heart failure (EF > 30%)** | 0 | 1 (0.28%) | 18 (6.82%) | 3 (11.11%) | 22 (3.22%) |
| **Heart failure (EF < 30%)** | 0 | 0 | 0 | 3 (11.11%) | 3 (0.44%) |
| **stroke < 3 months** | 0 | 0 | 2 (0.76%) | 0 | 2 (0.29%) |
| **stroke > 3 months** | 0 | 3 (0.84%) | 16 (6.06%) | 2 (7.41%) | 21 (3.07%) |
| **Moderate valve dysfunction** (insufficiency/stenosis I–II°) | 0 | 0 | 20 (7.58%) | 4 (14.8%) | 24 (3.51%) |
| **Severe valve dysfunction** (insufficiency/stenosis III°) | 0 | 0 | 0 | 0 |  |
| **Cardiac arrhythmia** | 0 | 8 (2.25%) | 32 (12.12%) | 12 (44.44%) | 52 (7.6%) |
| **Pacemaker/ICD** | 0 | 0 | 8 (3.03%) | 2 (7.41%) | 10 (1.46%) |
| **Anticoagulation** | 0 | 9 (2.53%) | 23 (8.71%) | 9 (33.33%) | 41 (5.99%) |
| **COPD** | 0 | 7 (1.97%) | 18 (6.82%) | 8 (29.69%) | 33 (4.82%) |
| **Other pulmonary diseases (asthma, OSAS)** | 0 | 26 (7.2%) | 13 (4.92%) | 1 (3.7%) | 40 (5.85%) |
| **Malignancy at time of surgery and/or previous to surgery** | 9 (24.32%) | 221 (62.08%) | 164 (62.12%) | 17 (62.96%) | 411 (60.09%) |
| **Malignancy at time of surgery, not metastasised** | 9 (24.32%) | 145 (40.73%) | 118 (44.7%) | 8 (29.63%) | 280 (40.94%) |
| **Malignancy at time of surgery, not metastasised** |  | 48 (13.48%) | 37 (14.02%) | 4 (14.81%) | 89 (13.01%) |
| **Previous malignancy** | 2 (5.41%) | 48 (13.48%) | 37 (14.02%) | 8 (29.63%) | 95 (13.89%) |
| **Haematological neoplasia (leukaemia)** | 0 | 6 (1.69%) | 5 (1.89%) | 0 | 11 (1.62%) |
| **Radio-/immuno-/chemo-therapy during 3 months prior to surgery** | 0 | 19 (5.34%) | 10 (3.79%) | 4 (14.81%) | 44 (6.4%) |
| **Decompensated liver cirrhosis** | 0 | 0 | 9 (3.41%) | 2 (7.41%) | 11 (1.62%) |
| **Active hepatitis** | 0 | 2 (0.56%) | 6 (2.27%) | 0 | 8 (1.17%) |
| **CKD** | 0 | 3 (0.84) | 23 (8.71%) | 6 (22.22%) | 32 (4.68%) |
| **CKD 5 (dialysis)** | 0 | 0 | 4 (1.52%) | 1 (3.7%) | 5 (0.73%) |
| **Diabetes mellitus (requiring/not requiring insulin)** | 0 | 48 (13.48%) | 60 (22.73%) | 6 (22.22%) | 114 (16.67%) |
| **Post-transplant** (kidney, heart, liver, stem cells) | 0 | 5 (1.4%) | 13 (4.92%) | 2 (7.41%) | 20 (2.92%) |
| **IBD** (ulcerative colitis, Crohn's disease) | 0 | 6 (1.69%) | 16 (6.06%) | 1 (3.7%) | 23 (3.36%) |
| **Immunosuppression** | 0 | 30 (8.43%) | 43 (16.29%) | 6 (22.22%) | 79 (11.55%) |
| **HIV** | 0 | 1 (0.28%) | 10 (3.79%) | 1 (3.7%) | 12 (1.75%) |
| **No systemic disease** | 33 (89.19%) | 5 (1.4%) | 0 | 0 | 38 (5.56%) |
| **Multimorbidity (≥ 2 systemic diseases)** | 0 | 62 (17.4%) | 162  (61.37%) | 22 (81.48%) | 246 (35.96%) |
| ASA-PS (American Society of Anaesthesiologists Physical Status Classification System); EF (ejection fraction); ICD (implantable cardioverter-defibrillator); COPD (chronic obstructive pulmonary disease); OSAS (obstructive sleep apnoea syndrome); CKD (chronic kidney disease); IBD (inflammatory bowel disease), HIV (human immunodeficiency virus) | | | | | |
