# Supplementary material for: Mind the gap: a prospective observational study of interprofessional differences in ASA-PS assessments between surgeons and anaesthesiologists
Source: BMC Anesthesiol. 2026 Feb 4;26:143. doi: 10.1186/s12871-026-03664-8 (PMC12934034; doi:10.1186/s12871-026-03664-8)
Supplement: Supplementary file 2 — Supplementary Material 2. [file 12871_2026_3664_MOESM2_ESM.docx]

**Supplementary Table 2** Frequency of minor and major postoperative complications.

| **Classification in minor and major complications** | **Distribution frequency** | **CDC I–V** | **Distribution frequency** |
| --- | --- | --- | --- |
| No evaluation | 8 (1.17%) | - | - |
| No complications | 396 (57.89%) | 0 | 396 (57.89%) |
| Minor complications | 138 (20.18%) | I | 80 (11.7%) |
|  |  | II | 58 (8.48%) |
| Major complications | 142 (20.76%) | IIIa | 34 (4.97%) |
|  |  | IIIb | 52 (7.6%) |
|  |  | IVa | 28 (4.09%) |
|  |  | IVb | 12 (1.75%) |
|  |  | V | 16 (2.34%) |
